# Seasonality, Annual Trends, and Characteristics of Dengue among Ill Returned Travelers, 1997–2006

**DOI:** 10.3201/eid1407.071412

**Published:** 2008-07

**Authors:** Eli Schwartz, Leisa H. Weld, Annelies Wilder-Smith, Frank von Sonnenburg, Jay S. Keystone, Kevin C. Kain, Joseph Torresi, David O. Freedman, for the GeoSentinel Surveillance Network

**Affiliations:** * Chaim Sheba Medical Center, Tel Hashomer, Israel; † Tel Aviv University, Tel Aviv, Israel; ‡ Statistical Consult, Victoria, British Columbia, Canada; § National University Singapore, Singapore; ¶ University of Munich, Munich, Germany; # Toronto General Hospital, Toronto, Ontario, Canada; ** University of Toronto, Toronto; †† University of Melbourne, Parkville, Victoria, Australia; ‡‡ Royal Melbourne Hospital, Parkville; §§ University of Alabama at Birmingham, Birmingham, Alabama, USA

**Keywords:** Dengue, travel, sentinel surveillance, seasonality, research

## Abstract

We examined seasonality and annual trends for dengue cases among 522 returned travelers reported to the international GeoSentinel Surveillance Network. Dengue cases showed region-specific peaks for Southeast Asia (June, September), South Central Asia (October), South America (March), and the Caribbean (August, October). Travel-related dengue exhibited annual oscillations with several epidemics occurring during the study period. In Southeast Asia, annual proportionate morbidity increased from 50 dengue cases per 1,000 ill returned travelers in nonepidemic years to an average of 159 cases per 1,000 travelers during epidemic years. Dengue can thus be added to the list of diseases for which pretravel advice should include information on relative risk according to season. Also, dengue cases detected at atypical times in sentinel travelers may inform the international community of the onset of epidemic activity in specific areas.

## Abstract 2

Atypical patterns may indicate onset of epidemic activity.

An estimated 100 million cases of dengue fever (DF) and 250,000 cases of dengue hemorrhagic fever (DHF) occur annually (*1*). The past 20 years have seen a dramatic geographic expansion of epidemic DF and DHF from Southeast Asia to the South Pacific Islands, the Caribbean, and the Americans. An increasing number of reports of DF and associated illness among travelers to dengue virus–infected areas parallel the changing epidemiology of dengue in local populations (*2*–*8*). As part of a comprehensive analysis of the spectrum of disease in travelers, the GeoSentinel Surveillance Network, a multifaceted international practice network, has noted that in terms of cumulative case numbers over the past decade, dengue has emerged as a more frequent diagnosis than malaria in ill travelers who have returned from all tropical regions outside of Africa (*9*,*10*).

Dengue accounts for up to 2% of all illness in returned travelers who visit GeoSentinel clinics (*9*). Dengue is also a major cause of hospitalization in febrile returned travelers (*7*,*11*). Prospective seroconversion studies have estimated the attack rate of DF in travelers to the tropics to be 2.9% in Dutch travelers who spent 1 month in Asia (*12*); the seroconversion rate was 6.7% among Israelis who traveled for an average of 5 months (*13*).

We report year-to-year variability, patient characteristics, travel exposures, and region/country specific proportionate illness rates due to dengue in 522 returned travelers. Our sample, collected over a decade, was also of sufficient size to examine the seasonality of dengue in travelers by region. Finally, the use of travelers as sentinels can help provide timely information to the international community about the onset of dengue outbreaks in disease-endemic areas.

## Methods

### GeoSentinel Surveillance Network

GeoSentinel sites are specialized travel/tropical medicine clinics on 6 continents staffed by clinicians who are recruited on the basis of demonstrated training, experience, and publication in travel and tropical medicine literature. They contribute clinician-based information on all ill travelers seen, including travel history (additional detail is available from www.geosentinel.org) (*9*,*14*). The sites that account for most patient intake are within academic centers; several smaller volume sites (almost all with current academic affiliation) are in freestanding locations. The intake at sites reflects a mixed population of tertiary care and self-referred patients. Some sites are restricted to outpatients, and no one site limits its entire practice to ill travelers. To be eligible for inclusion in the GeoSentinel database, patients must have crossed an international border and be seeking medical advice at a GeoSentinel clinic for a presumed travel-related illness. Anonymous surveillance data that cannot be linked to an individual patient are entered into an SQL database at a central data center. Final diagnoses reported by physicians are used to assign diagnostic codes from a standardized list of >500 etiologic or syndromic diagnoses (*9*).

### Inclusion/Exclusion Criteria

All returning travelers who reported to a GeoSentinel site in their current country of residence from October 1, 1997, to March 1, 2006, were eligible for analysis. Many GeoSentinel sites also enter data separately on immigrants with no other travel but the initial immigration trip. None of the patients in this immigrant dataset had a diagnosis of dengue acquired in the country of origin. The current study is restricted to traditional travelers, which also includes immigrants who subsequently traveled from their current country of residence.

Patients were excluded if no confirmed or probable diagnosis was reported. A case of travel-associated dengue was defined per current annual surveillance reports (*15*–*17*), which consider both probable and confirmed cases of dengue (*18*). A case of travel-associated dengue was defined as laboratory-diagnosed dengue in a resident of a non–dengue-endemic area who has traveled to a dengue-endemic area in the 14 days before symptom onset. Laboratory-diagnosed dengue was determined by isolation of dengue virus, virus antigen, or viral RNA, or a serum sample positive for either immunoglobulin (Ig) M or a very high titer of IgG by ELISA. All sites use best available reference diagnostics for their respective countries, which may include well-characterized commercial kits. GeoSentinel criteria for the diagnosis of malaria have been reported (*19*).

### Statistical Analysis

Analysis of dengue reports over time was based on proportionate morbidity (the number of patients with dengue fever as a proportion of the number of ill returned travelers visiting a GeoSentinel clinic in that month). Analysis of annual and monthly cycles was based on monthly proportionate morbidity aggregated over all years of data included in the analysis. Patients who were reported as having dengue were compared with all other ill returned travelers in GeoSentinel. A subanalysis, comparing dengue patients with malaria patients, was also performed. We used χ^2^ or Fisher exact test as appropriate with a 2-sided significance level of 0.05. Data analysis was performed by using SAS statistical package version 9 (SAS Institute, Cary, NC, USA).

## Results

Among ill returned travelers seen at GeoSentinel sites from October 1997 through February 2006, 24,920 met the criteria for analysis. Of these, 522 (2.1%) had a diagnosis of travel-related dengue fever, including 12 patients with dengue hemorrhagic fever or dengue shock syndrome. Of the 522 cases of dengue reported in this study, 68% were seen after travel to Asia, 15% after travel to Latin America, 9% after travel to the Caribbean, 5% after travel to Africa, and 2% after travel to Oceania (Table 1). The countries with the largest number of cases reported among returned travelers were Thailand (154), India (66), Indonesia (38), and Brazil (22).

**Table 1:** Dengue and malaria diagnoses as a proportion of all morbidity in ill returned travelers according to region or country of acquisition

Region* or country of exposure	No. ill returned travelers with dengue	No. ill returned travelers with malaria	Total no. ill returned travelers	Dengue proportionate morbidity†	Malaria proportionate morbidity†
Southeast Asia	264	103	3,694	71	28
Thailand	154	9	1,523	101	5
Indonesia	38	53	652	58	81
South Central Asia	90	70	3,303	27	21
India	66	57	2,119	31	27
Caribbean	47	14	1,470	32	9
South America	40	49	2,427	16	20
Brazil	22	12	685	32	18
Central America	37	27	1,867	20	14
Africa	25	1,216	7,231	3	168
Sub-Saharan Africa	23	1,201	6,201	4	194
Oceania	11	91	303	36	300
Other‡ or multiple regions of exposure	7	23	4,443	2	5
Country missing	1	12	182	5	66
Total	522	1,605	24,920	21	64

*Regions defined per (*9*). †Proportionate morbidity expressed per 1,000 ill returned travelers seen at GeoSentinel clinics. ‡No cases were acquired in Canada, United States, Western Europe, Japan, or Australia.

### Annual Trends in Travel-related Dengue and Changes during Regional Epidemics

**Figure 1:**
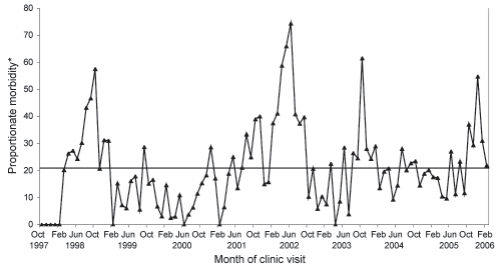
Dengue fever in returned travelers as a proportion of monthly morbidity in all ill returned travelers to all regions of the world. Cumulative proportionate morbidity of 21 per 1,000 ill returned travelers (522 dengue reports among 24,920 ill travelers from October 1997 through February 2006) is shown by the horizontal line. *Proportionate morbidity is expressed as number of dengue cases per 1,000 ill returned travelers.

A comparison of the annual trends in illness from dengue as a proportion of all diagnoses in ill returned travelers showed sustained increases in dengue proportionate morbidity, represented by peaks that are both high and broad in 1998 and 2002. There was also a narrow peak in October 2003 and an increase in late 2005 (Figure 1). When dengue reports were segregated by region, the increases in 1998 and 2002 were found entirely in travelers to Southeast Asia; for 2003, in travelers to South Central Asia; and for 2005, in travelers to South Central Asia and Indonesia. These increases correspond to known epidemic years within local populations for those regions (*20*,*21*).

**Figure 2:**
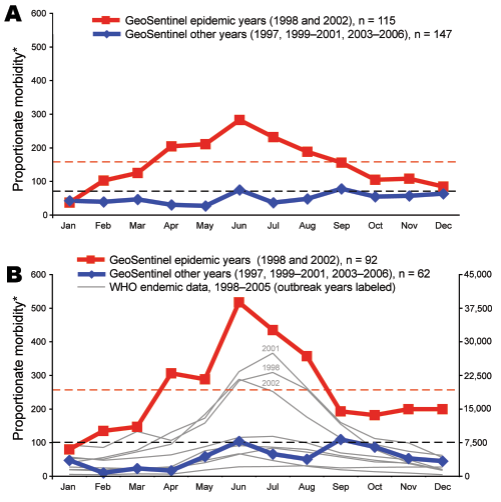
Changes in dengue morbidity during regional epidemics. Heavy red and blue lines show dengue in returned travelers as a proportion of monthly morbidity in all ill returned travelers to Southeast Asia (A) and Thailand (B) during the epidemic years of 1998 and 2002 (red lines) and during all other nonepidemic years (blue lines). Black horizontal dashed lines represent mean proportionate morbidity over all months for that area during the cumulative 1997–2006 period in travelers; red horizontal dashed lines represent mean proportionate morbidity over all months during the 2 outbreak years (1998 and 2002) in travelers. Each gray line in panel B tracks month-by-month reports to the World Health Organization (WHO) of the total number of dengue cases in the endemic Thai population for a single year from 1998–2005. *Proportionate morbidity is expressed as number of dengue cases per 1,000 ill returned travelers.

Since travel-related dengue was found to originate most commonly in Southeast Asia, more detailed analysis could be performed for that region. Dengue proportionate morbidity among ill returned persons who had traveled to Southeast Asia, which was a mean of 71 per 1,000 during the cumulative 1997–2006 period (Figure 2, panel **A**), was 159 cases per 1,000 ill returned travelers during 1998 and 2002 taken together (Figure 2, panel **A**); proportionate morbidity reached a peak of >200 cases per 1,000 ill returned travelers during June and July. Of the 264 Southeast Asian cases, 154 that were acquired in Thailand could be plotted separately (Figure 2, panel **B**). Dengue proportionate morbidity among ill persons who had traveled to Thailand, which was an average of 101 cases per 1,000 during the cumulative 1997–2006 period (Figure 2, panel **B**), was 257 cases per 1,000 ill returned travelers during 1998 and 2002 taken together (Figure 2, panel **B**) and was >500 cases per 1,000 ill returned travelers during the peak month of June (i.e., more than half of all ill travelers returning from Thailand had dengue).

### Seasonality of Travel-associated Dengue

**Figure 3:**
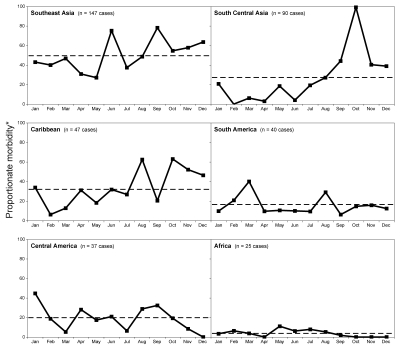
Seasonality of dengue in returned travelers by region. Dengue in returned travelers is shown as a proportion of monthly morbidity in all ill returned travelers to each region. Horizontal dashed lines represent the mean proportionate morbidity over all months for that region during the cumulative 1997–2006 period in travelers. Data for Southeast Asia exclude the outbreak years of 1998 and 2002. *Proportionate morbidity is expressed as number of dengue cases per 1,000 ill returned travelers.

Figure 3 shows month-by-month dengue cases as a proportion of all illness in ill returned travelers during the study period for each region separately. For Southeast Asia, dengue cases generally peaked in June and September in typical nonepidemic years. However, an examination of the outbreak years of 1998 and 2002 showed that seasonal patterns changed markedly when compared with nonoutbreak years; excess cases were seen for every month except January, and a high and sustained peak occurred from April through August (Figure 2, panel **A**). In Thailand, during the outbreak years, proportionate morbidity exceeded the mean 1997–2006 proportionate morbidity (Figure 2, panel **B**) for all months except January. Notably, the major peak of illness began in April, a time of minimal dengue activity in nonoutbreak years. The major epidemic peak in sentinel travelers preceded the epidemic pattern in the local population during 1998 and 2002, as reflected in Thai reports to the World Health Organization (*20*).

An examination of seasonality in travelers for other regions (Figure 3) showed that dengue cases were higher from September through December in South Central Asia (especially in India, which accounted for most of our cases in South Central Asia; Table 1). A sharp and consistent major peak can be seen each October throughout the study period. This closely tracks the monthly pattern in the Indian population during 2003–2006, years for which robust Indian national data are available (*21*). The number of dengue cases in travelers was higher from August through December in the Caribbean; it was highest in March in South America, especially in Brazil, which accounted for most of our cases in South America (Table 1). This peak is also consistently seen in the Brazilian population (*22*) each year. No evidence of a strong seasonal pattern was found in travelers to Central America and Africa, although the numbers of travelers to these regions in our sample were small.

### Characteristics of Travelers with Dengue

The characteristics of travelers with dengue are compared in Table 2 with the characteristics of those with all other diagnoses. The mean age of dengue patients was 33.8 years; the male:female ratio was 1.17:1. Of the patients studied, 69.3% were traveling only for tourism, and the median trip duration was <28 days. Most of the dengue case-patients (61%) had a pretravel encounter, a significantly higher percentage than for ill returned travelers without dengue (53%; p<0.005). Dengue is overwhelmingly a disease of young adults 18–44 years of age. As expected, due to the short incubation period, >75% of dengue case-patients sought treatment within 2 weeks after return. In addition, significantly more dengue patients were hospitalized (24% vs. 6%; p<0.001), a level similar to the 25% rate reported in a study of European travelers (*4*).

**Table 2:** Demographic characteristics and type of travel for ill returned travelers with dengue, malaria, or any other diagnosis

Characteristic	Ill returned travelers with dengue	Ill returned travelers with malaria	Ill returned travelers without dengue or malaria	Total ill returned travelers
No. cases	522	1,605	22,793	24,920
Age group, %*				
<18 y	1.3	5.6	3.9	3.9
18–44 y	79.2	68.4	69.1	69.3
45–55 y	12.4	17.2	14.7	14.7
>56 y	7.1	8.9	12.4	12.0
Female sex, %*	46.1	30.1	49.7	48.3
Pretravel encounter, %*				
Yes	61.3	42.8	53.6	53.1
No	28.6	43.7	33.6	34.1
Unknown	10.1	13.5	12.8	12.8
Inpatient, %*	24.8	50.3	6.6	9.8
Duration of travel, d*				
25% travelers	14 d	21 d	14 d	14 d
50% travelers	28 d	34 d	28 d	28 d
75% travelers	67 d	95 d	132 d	123 d
Time from travel to symptoms, %*				
<2 week	75.5	53.3	41.8	43.3
>2 week	24.5	46.7	58.2	56.7
Patient classification %*				
Immigrant	7.9	35.1	13.6	14.9
Temporary visitor	4.2	2.2	1.6	1.7
Expatriate	8.4	13.7	10.0	10.2
All other travelers	79.5	48.4	74.5	73.1
Reason for recent travel, %*				
Tourism	69.3	29.1	60.3	58.5
Business	10.5	16.0	14.0	14.1
Research/education or Missionary/volunteer	10.2	14.5	12.7	12.8
Visiting friends or relatives†	9.8	40.1	12.5	14.2

*Significant differences in travelers with dengue vs. malaria (p<0.05). †1st or 2nd generation immigrant originally from a low-income country now living in a high-income country, visiting friend and relatives in the country of the family’s origin.

### Comparison of Dengue and Malaria Patient Characteristics

During the study period, 1,605 (6.4%) ill returned travelers reported to GeoSentinel had been given a diagnosis of malaria. A comparison of the characteristics of travelers with dengue and those with malaria shows some important differences. Unlike dengue, which affects both sexes almost equally, malaria is more common in male travelers (*11*,*23*). Patients with malaria were less likely to have a recorded pretravel encounter. Duration of travel (median 34 days) was significantly longer than for travelers returning with dengue (median 28 days; p<0.05), although the difference was not large. Malaria was much more common in first- or second-generation immigrants visiting friends and relatives (Table 2).

Overall, the proportion of travelers with dengue in the GeoSentinel database (21 cases per 1,000 ill returned travelers) was less than the proportion seen with malaria (64 cases per 1,000 ill returned travelers; Table 1). This finding, however, was mostly due to the disproportionate numbers of travelers returning ill from Africa where malaria is highly prevalent (168 cases per 1,000 ill returned travelers) and where dengue is rare (3 cases per 1,000 ill returned travelers). A similar situation applies to Oceania where malaria (300 cases per 1,000 ill returned travelers) is significantly more frequent than dengue fever (36 cases per 1,000 ill returned travelers). For other regions, the proportionate morbidity due to dengue was higher than that due to malaria, except for South America, where proportionate morbidity was approximately equal (20 cases vs. 16 cases per 1,000 ill returned travelers).

Analysis of travel to several countries was possible. Dengue proportionate morbidity (101 cases per 1,000 ill returned travelers) was dramatically higher than that for malaria (5 cases per 1,000 ill returned travelers) in travelers returning from Thailand and exceeded that for malaria in travelers returning from Brazil and India.

## Discussion

Data collected longitudinally over a decade by the GeoSentinel Surveillance Network have allowed us to examine month-by-month illness from a sample of 522 patients with dengue (as a proportion of all diagnoses among 24,920 ill returned travelers) seen at our 33 surveillance sites. Travel-related dengue demonstrates defined seasonality for some regions (Southeast Asia, South Central Asia, the Caribbean, and South America; Figure 3). Although discrete peaks are present, the number of cases from the Caribbean and South America is relatively small. A June peak of travel-related dengue was previously reported in a small sample of 75 Swedish travelers to Thailand (using imported cases from 1998–1999) (*3*). Several vector-borne diseases, such as malaria (*24*) and Japanese encephalitis (*25*), are known to exhibit seasonality in local populations, but no firm data exist on whether this pertains to travelers’ risk. Our findings on the seasonality of dengue in travelers benefits those advising prospective travelers, as well as those formulating possible diagnoses in ill returned travelers. Consequently, travelers who have had a previous episode of dengue might want to avoid peak dengue transmission times at a particular destination to minimize the risk for developing dengue hemmorhaghic fever, which preferentially affects those with previous dengue infection (*26*). For example, the February–March peak in Brazil coincides with Carnaval (annual festival marking the beginning of Lent). Nevertheless, in dengue-endemic regions, risk exists year round, and travelers should always be counseled on personal protection measures against arthropods.

Rainy seasons vary by country and, in many cases, vary regionally within countries. Because of these geographic variations in the rainy season, we have avoided the temptation to over generalize about relationships between rainfall and dengue incidence (Figure 3). Although GeoSentinel would not be likely to receive reports from outbreaks of dengue that are restricted to regions of a country not frequented by travelers, most substantial outbreaks do eventually spread widely (*27*). In this analysis, proportionate morbidity always compares the number of dengue cases with all ill travelers seen at GeoSentinel clinics during a particular month. This type comparison ensures that the variation in the absolute number of travelers to a particular destination at different times of year do not distort the results.

The natural, and to a large extent unexplained, year-to-year oscillations of dengue cases in local populations have been described in some countries (*27*–*29*). In travelers, this has not been examined over a long period in such a sizeable dataset, while simultaneously comparing regions of the world (*30*). In each of the epidemic years 1998 and 2002 in Southeast Asia, the usual pattern of seasonality changed with an excess of cases throughout the whole year. The outbreak was heralded initially by an excess of cases beginning in February with a dramatic upsurge in April (Figure 2, panel **A**), well ahead temporally and in magnitude when compared with the usual initial peak month of June. When the 1998 pattern in travelers recurred in early 2002, it led to the immediate hypothesis that this change of seasonality would once again herald an epidemic year. In April 2002, GeoSentinel alerted the international community when it posted a notice of the increase in travel-related dengue from Thailand online (*31*). Official surveillance data from local populations were not immediately available to the international community. Data reported later by Thai authorities to the World Health Organization confirmed the observation (*20*). A retrospective report published in 2004 also noted an April 2002 surge in dengue cases among German travelers to Thailand (*32*). The increase in dengue cases in returned travelers from South Central Asia in 2003 was also evident before official surveillance data were available. This increase reinforces the usefulness of sentinel surveillance in travelers. For example, travelers’ malaria has identified new foci of infection in the Dominican Republic (*33*) and the Bahamas (*34*). Because the number of travelers to areas with epidemics may be small and some epidemics may occur in parts of a country that are not visited by travelers, we are not proposing sentinel surveillance as a definitive and uniquely sensitive tool for detection of all disease outbreaks. A 2001 outbreak in Thailand apparently did not affect travelers (Figure 2, panel **B**), as it was not associated with a peak in reports to GeoSentinel. Nevertheless, the traveling population can give timely, very specific indicators.

Our data on the high frequency of dengue in travelers to Southeast Asia and the Caribbean and its rarity in travelers to Africa are in agreement with previous smaller samples such as those from a regional European surveillance network (TropNetEurop), which examined 238 returning travelers with dengue over a 3-year period (1999–2001) (*4*). In comparing proportionate morbidity for dengue between regions, rates in travelers to the Caribbean approach those of some parts of Asia and are thus higher than would be expected from overall rates in local populations. These rates likely reflect common travel patterns that may favor more risky locales. A new finding in our report is the high proportionate morbidity in travelers to Oceania, who because of small absolute numbers of travelers to that region, may have been overlooked in earlier studies, which reported only raw numbers of cases.

The limitations of this analysis include those applicable to other published studies that used the GeoSentinel database. The findings can only be generalized to travelers seen in tropical or travel medicine clinics after travel. In general, data do not represent a sample of all returned travelers (e.g., those seen at nonspecialized, primary care practices, where milder and self-limited manifestations of dengue that might not be recognized as such, would occur with greater frequency). The more severely ill patients that do seek treatment at specialized clinics such as GeoSentinel sites will likely have higher hospitalization rates than the overall population with dengue infection. Patients may also seek treatment at GeoSentinel sites and not return for follow-up definitive diagnostic serology when faced with the inconvenience and cost of serologic evaluation of a self-limited illness, particularly when symptoms have resolved. Dengue has a short incubation period; many patients may have the disease while still traveling. Nevertheless, the uncaptured cases are not likely to have a different pattern of geographic acquisition than those that are included.

In conclusion, current data serve as a reference for the seasonality of dengue for several regions of the world. Dengue can be added to the list of diseases for which pretravel advice can include information on relative risk according to season of travel to a particular destination. Further, the season of travel can aid the clinician in assessing the relative likelihood of dengue in an ill returned traveler with a nonspecific febrile illness. Travelers may be sentinels able to rapidly inform the international community about the onset of epidemics in disease-endemic areas. Effective malaria chemoprophylaxis and strategies for personal protection against night-feeding malaria vectors are already available. Dengue is a risk for all tourists equally without respect to gender, pretravel preparation, or duration of travel. Even with good pretravel advice, all healthcare providers can do is recommend mosquito precautions. The usual preventive measure for an infectious disease is vaccination. Because personal protection against the day-feeding dengue vectors is so problematic, there is an urgent need for a dengue vaccine**.**
